# The role of IGF2BP2, an m6A reader gene, in human metabolic diseases and cancers

**DOI:** 10.1186/s12935-021-01799-x

**Published:** 2021-02-10

**Authors:** Jinyan Wang, Lijuan Chen, Ping Qiang

**Affiliations:** 1 Department of Oncology, Zhangjiagang First People’s Hospital, Zhangjiagang Affiliated Hospital of Soochow University, Zhangjiagang, China; 2 The Affiliated Jiangning Hospital of Nanjing Medical University, Nanjing, China; 3 Department of Gynecology, Zhangjiagang First People’s Hospital, Zhangjiagang Affiliated Hospital of Soochow University, Zhangjiagang, 215600 Jiangsu People’s Republic of China

**Keywords:** IGF2BP2, m6A, Metabolic disease, Cancers, Biological function

## Abstract

The human insulin-like growth factor 2 (IGF2) mRNA binding proteins 2 (IGF2BP2/IMP2) is an RNA-binding protein that regulates multiple biological processes. Previously, IGF2BP2 was thought to be a type 2 diabetes (T2D)-associated gene. Indeed IGF2BP2 modulates cellular metabolism in human metabolic diseases such as diabetes, obesity and fatty liver through post-transcriptional regulation of numerous genes in multiple cell types. Emerging evidence shows that IGF2BP2 is an N6-methyladenosine (m6A) reader that participates in the development and progression of cancers by communicating with different RNAs such as microRNAs (miRNAs), messenger RNAs (mRNAs) and long non-coding RNAs (lncRNAs). Additionally, IGF2BP2 is an independent prognostic factor for multiple cancer types. In this review, we summarize the current knowledge on IGF2BP2 with regard to diverse human metabolic diseases and its potential for cancer prognosis.

## Introduction

**Fig. 1:**
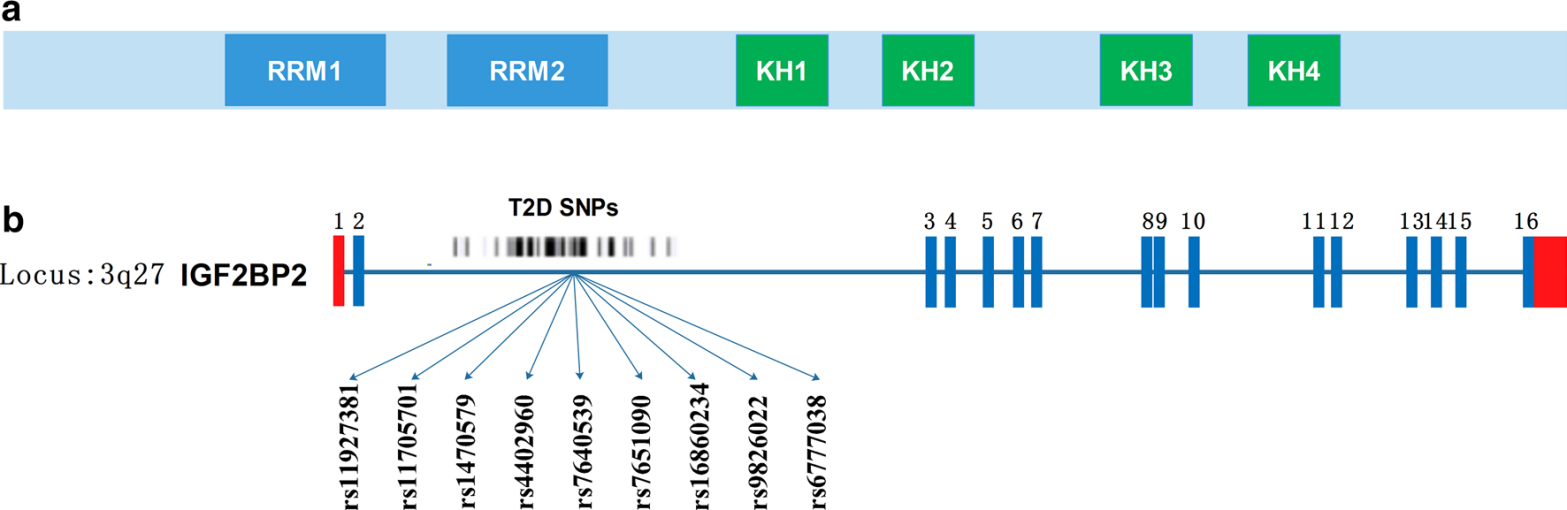
**a** The structure of RRMs and KH domains. **b** Polymorphisms in the IGF2BP2. Boxes represent exons whereas the red color represents 5′ and 3′ untranslated regions of IGF2BP2. The ~ 80 T2D-associated SNPs are localized within the second intron of the IMP2 gene at chromosome 3

The human insulin-like growth factor 2 (IGF2) mRNA binding proteins (IMP1-3 or IGF2BP1-3), first identified in 1999, attaches to the 5′ untranslated regions (5′ UTRs) of the translationally regulated IGF-II reader mRNA [1]. IGF2BP2, with a molecular mass of 66 kDa, has two N-terminal RNA-recognition motifs (RRMs) and four C-terminal human heterogeneous nuclear ribonucleoprotein (hnRNP)-K homology (KH) domains [1, 2] (Fig. 1a). Expression of IGFBP2 is generally maintained postnatally and participates in localization, stability and translation of RNAs [3].

Recent genome-wide association studies (GWAS) have revealed that IGF2BP2 gene induces the development of type 2 diabetes (T2D) by disrupting insulin secretion [2]. Mechanistically, IGF2BP2 modulates cellular metabolism by post transcriptional regulation of several genes in numerous cell types and pathways [4]. In addition, dysregulation of IGF2BP2 is associated with progression of cancers and cancer stem cells [5]. Recently, IGF2BP2 has been shown to read N6-methyladenosine (m6A), the most abundant internal RNA modification in eukaryotic cells [6]. IGF2BP2 communicates with several RNAs such as microRNAs (miRNAs) [7], messenger RNAs (mRNAs) [8] and long non-coding RNAs (lncRNAs), where it regulates several biological processes [9]. M6A-RNA methylation refers to methylation of adenosine bases at position N6 in 3′UTRs near the stop codons but within the internal long exons [10, 11]. Modified IGF2BP2 participates in the development and progression of multiple metabolic disease and cancers, including diabetes [2], obesity [12], fatty liver [13], breast cancer [14], colorectal carcinoma [15], esophageal adenocarcinoma [16], glioma [17], hepatocellular carcinoma [18], lung cancer [19], pancreatic cancer [20] and many others.

In this review, we summarize the current evidence on the relationship between IGF2BP2 and metabolic disease, as well as the biological mechanisms underlying IGF2BP2 functions in cancers.

## Expression of IGF2BP2 and metabolic disease

Murine models have uncovered the role of IGF2BP2 in metabolic diseases including diabetes, obesity, fatty liver and among others [4]. Based on the GWAS, a cluster of single nucleotide polymorphisms (SNPs) in the second intron of IGF2BP2 (Fig. 1b) have been implicated for T2D. The association between IGF2BP2 and human metabolic diseases stems in its post-transcriptional regulation of numerous genes in different cell types and pathway [4]. The sections that follow discuss in detail, the associations between IGF2BP2 and several metabolic diseases (Table 1).

**Table 1:** The roles of IGF2BP2 in various metabolic diseases and the associations between the expression of IGF2BP2 SNPs and the development several metabolic diseases as well as cancer in different populations

Metabolic diseases	SNPs	Population	Biological functions	Refs.
Diabetes	rs4402960	Chinese Han population	Protected against T2D, enhanced the therapeutic efficacy of repaglinide, and reduced the effect of pioglitazone on PPG, TG, and HDL-C	[27–31, 41, 42]
		Japanese population	\	[32]
		Asians	\	[27, 33]
		Iceland’s population	Decreased fasting insulin levels, impaired β-cell function	[34]
		Greek-Cypriot population	\	[35]
		Czech population	\	[26]
		Germany population	\	[33, 36]
		Lebanese Arabs	\	[37]
		Arab population	\	[38]
		Moroccan population	\	[38]
		Tunisian population	\	[39]
		India’s population	\	[40]
		\	Predict the occurrence and diagnosis of GDM	[43]
		Poland population	Influenced the length of gestation and the Apgar scores of newborns	[44]
	rs1470579	Chinese Han population	Reduced the therapeutic efficacy of repaglinide and the effect of pioglitazone on PPG, TG, and HDL-C	[29, 30, 42]
		Lebanese population	\	[46]
		Iranian population	\	[47]
	rs11705701	Mexican American population	Affected insulin resistance	[53]
		Russian population	Contributed to T2D risk, decreased levels of p58 and increased levels of p66 of the IGF2BP2 in adipose tissue of non-obese individuals	[51]
		Poland population	Influenced the length of gestation and the Apgar scores of newborns	[44]
		\	Associated with prediabetes	[54]
	rs9826022	\	\	[48]
	rs11927381	Chinese Han population	\	[55]
	rs7640539			[55]
	rs6777038	\	Associated with GADA negative diabetes	[56]
	rs16860234			
	rs7651090			
Nonalcoholic steatohepatitis	\	\	Increased the ratio of C18:C16 and the expression of ELOVL6	[63]
	\	\	Promoted the de-differentiated cells toward steatohepatitis-associated cirrhosis development via accelerating DR	[64]
Obesity	\	\	IGF2BP2 deficiency induced the resistance to diet-induced obesity and fatty liver, and showed great glucose tolerance and insulin sensitivity	[12]
Fatty liver	\	\	IGF2BP2 knockout impaired fatty acid oxidation and promoted modest diet-induced fatty liver	[13]

“\”: indicates not mentioned

*SNP* single nucleotide polymorphisms, *T2D* type 2 diabetes, *PPG* postprandial plasma glucose, *TG* triglycerides, *HDL-C* high-density lipoprotein cholesterol, *GDM* gestational diabetes mellitus, *GADA* glutamic acid decarboxylase antibodies, *ELOVL6* fatty acid elongase 6, *DR* ductular reaction

### Expression of IGF2BP2 and diabetes

In 2007, Grarup, et al. [21] found no association between IGF2BP2 genetic variants and pancreatic-cell dysfunction in Danish population. Later on, the IGF2BP2 variant was found to decrease glucose-stimulated insulin secretion in the first but not the second phase of diabetes development [22]. Additionally, IGF2BP2 has also been implicated in the development of T2D/impaired glucose tolerance [23]. In Indian [24] and Chinese [25] populations, IGF2BP2 was found to be closely associated with T2D even after adjusting for age, sex and BMI. For European, Czech and Swedish populations, IGF2BP2 polymorphism has been associated with diabetic nephropathy in male patients with type 1 diabetes (T1D) [26]. Accordingly, we summarize how IGF2BP2 SNPs participate in the development of diabetes.

#### Expression of IGF2BP2 rs4402960 and rs1470579 in diabetes

IGF2BP2 rs4402960 and rs1470579 are the most common SNPs in diabetes. Research shows that expressions of IGF2BP2 rs4402960 gene variant in Chinese Han [27–31], Japanese [32], Asian [27, 33], Icelandic [34], Greek-Cypriot [35], Czechs [26], Germania [33, 36], Lebanese [37], Arabian [38], Tunisian [39], Moroccan [38] and Indian population [40] increase the risk for T2D. IGF2BP2 rs4402960 is also associated with lower fasting insulin level and impaired β-cell function, both associated with obesity [34]. Meanwhile, wild C IGF2BP2 rs4402960 allele protects against T2D in Chinese Han individuals. In addition, the therapeutic efficacy of repaglinide is enhanced in Chinese T2D patients with IGF2BP2 rs4402960 polymorphism [41]. The effect of pioglitazone on postprandial plasma glucose, glycated hemoglobin, serum triglycerides and high-density lipoprotein cholesterol is in Chinese individuals with rs4402960 polymorphism [30]. Moreover, IGF2BP2 rs4402960 is strongly associated with the development of gestational diabetes mellitus (GDM), besides being a potential diagnostic marker for GDM as well [42]. However, no association has been found between IGF2BP2 rs4402960 polymorphism and the risk of developing GDM in Polish [43] and Chinese [44] population, but it influence the length of gestation period and health (based on Apgar scores) of newborns in these populations.

On the other hand, IGF2BP2 rs1470579 is also associated with T2D in Lebanese [45], Chinese Han [29, 30] and Iranian populations [46]. In addition, IGF2BP2 rs1470579 polymorphism reduces the therapeutic efficacy of repaglinide in T2D patients in Chinese population [41]. The effect of pioglitazone against PPG, TG and HDL-C is also lower in Chinese patients with rs1470579 gene variant [30].

However, other researches failed to replicate the confirmed rs4402960 and rs1470579 susceptibility variants in French Caucasians [47], Indian [48, 49], Chinese Han [50] and Russian populations [51]. A global meta-analysis of 35 studies encompassing 175,965 subjects on the association between IGF2BP2 rs4402960 and rs1470579 and T2D revealed that even though these polymorphisms increases the risk of developing T2D, the associations vary among ethnic populations [52].

#### Other IGF2BP2 SNPs in diabetes

IGF2BP2 rs11705701 has been associated with low body fat, which contributes to insulin resistance and consequently T2D risk in Mexican American population [53]. IGF2BP2 rs11705701 has also been associated with higher risk of T2D in Russian population. Meanwhile, allele A of rs11705701 has been linked with low levels of short isoform (p58) but high levels of the long isoform (p66) of IGF2BP2 protein in adipose tissue of non-obese individuals [51]. Additionally, IGF2BP2 rs11705701 has been strongly associated with prediabetes in female patients [54]. Although no association has been found between IGF2BP2 rs11705701 and the risk of developing GDM in Polish population, it lengthens the gestation and improves the health (based on Apgar scores) of newborns in this population [43]. On the other hand, rs9826022, a rare mutation in the 3′downstream region of IGF2BP2, is closely associated with T2D [47]. Besides, IGF2BP2 rs11927381 and rs7640539 are all associated with the risk of developing T2D among Chinese Han population [55]. Meanwhile, rs6777038, rs16860234 and rs7651090 of IGF2BP2 are closely linked with glutamic acid decarboxylase (GAD) antibody- negative diabetes [56].

#### The mechanisms underlying IGF2BP2 in diabetic nephropathy

**Fig. 2:**
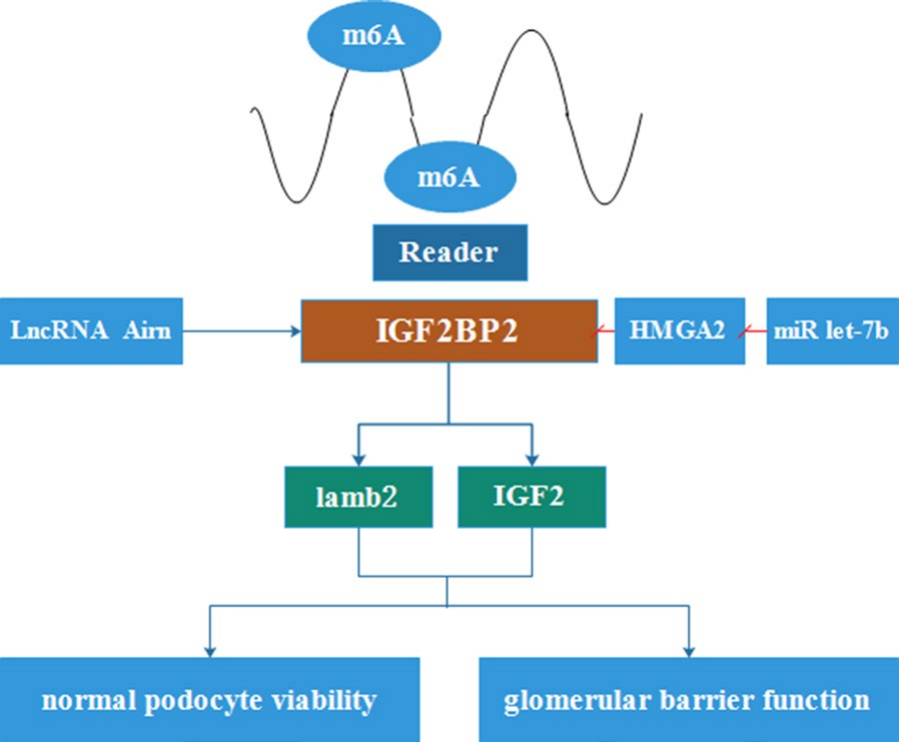
The role of IGF2BP2 in DN. IGF2BP2, regulated by *AIRN* and miR-let-7b/HMGA2, promotes proliferation of podocytes and functioning of the glomerular by targeting lamb2 and IGF2

Diabetic nephropathy (DN) is one of the most serious microvascular complications that increases the risk of death of T2D patients [57]. Recently, it is has been found that the role of IGF2BP2 in DN depends on interlinked communication with several other genes, miRNAs and lncRNAs (Fig. 2). Laminin-β2 (lamb2), the key laminin subunit, participates in maintaining normal basement membrane structure and function of the glomerular [58]. Intriguingly, IGF2BP2 regulates expression of lamb2 by directly targeting lamb2 mRNA in actin cytoskeleton [59]. IGF2 also regulates the regeneration and survival of podocytes [60, 61]. For instance, Jing, et al. [62] found that antisense of insulin-like growth factor-2 receptor non-coding RNA (*AIRN*) regulates the translation of IGF2 and lamb2 by binding to IGF2BP2, thus maintaining normal podocyte viability and glomerular barrier function, preventing DN. As such, *AIRN* is potentially a new therapeutic target against diabetic nephropathy in individuals with low lamb2 levels.

### IGF2BP2 in fatty liver and steatohepatitis and the specific mechanisms

IGF2BP2 up-regulates the expression of fatty acid elongase 6 (ELOVL6), which catalyzes the elongation of C16 fatty acids to C18, contributing to the development of human nonalcoholic steatohepatitis [63]. Besides, the activation of ELOVL6 is regulated by sterol regulatory element binding transcription factor 1. IGF2BP2 promotes the development of steatohepatitis-associated cirrhosis in de-differentiated cells by accelerating ductular reaction [64]. Dai et al. [12] found that IGF2BP2^−/−^ mice were highly resistant to diet-induced obesity and fatty liver disease, and showed greater glucose tolerance and insulin sensitivity. IGF2BP2 inhibits the translation of the untranslated Ucp1 bearing mRNAs and binds to mitochondrial components. However, Regué et al. [13] reported that specific IGF2BP2-hepatocyte knockout results in greater accumulation of triglycerides in the liver. This suggests that the expression of IGF2BP2, which encodes carnitine palmi toyltransferase 1A (CPT1A) and peroxisome proliferator-activated receptor, disrupts fatty acid oxidation, thus promoting accumulation of the fatty acids in the liver.

## IGF2BP2 and cancers

In recent years, over-expression of IGF2BP2 in multiple human cancers has been associated with poorer prognosis of the disease. For instance, over-expression of IGF2BP2 confers shorter survival and poor prognosis of patients with acute myelocytic leukemia (AML) [65], breast cancer [14], esophageal carcinoma [16], low-grade gliomas [17], hepatocellular carcinoma (HCC) [66], head and neck squamous cell carcinoma (HNSCC) [67], pancreatic ductal adenocarcinoma (PDAC) [20, 68–70] and gallbladder carcinoma (GBC) [71]. Herein, we summarize the specific roles of IGF2BP2 in multiple cancers and provide a comprehensive view of IGF2BP2 (Fig. 2; Table 2).

**Table 2:** The expression, clinical significance and biological functions of IGF2BP2 in different cancer types

Cancer	Expression	Role	Biological function	Upstream	Target	Refs.
AML	↑	Oncogene	Cell growth		/	[65]
Breast cancer	/	Oncogene	Proliferation, invasion	miR-1193	ERK, PI3K/Akt	[7]
	↑	Oncogene	/	/	/	[14]
	↑	Oncogene	Autoantibody response	/	/	[73]
	↑	Oncogene	Tumor growth	CCN6	/	[74]
Colorectal cancer	↑	Oncogene	Proliferation, survival	/	miR-195/RAF1	[81]
	/	Oncogene	Invasion, proliferation, migration, MDV, EMT, apoptosis	LncRNA HOTAIR	/	[9]
	/	Oncogene	Glycolysis, proliferation	LINRIS	MYC	[100]
	/	/	Proliferation, migration, invasion, autophagy	91H	IGF2	[103]
	/	/	Cell self-renewal, stem cell frequency, migration, tumorigenesis, metastasis	METTL3	SOX2	[15]
Glioma	/	/	Proliferation, migration, invasion	miR-188	/	[91]
	↑	Oncogene	Proliferation, invasion	miR-138	/	[17]
HCC	↑	Oncogene	Proliferation, migration, invasion	miR-216b	/	[92]
	/	Oncogene	Proliferation, metastasis	lncRNA RHPN1-AS1/miR-596	/	[104]
	/	/	/	MIRLRT7A3/miR-let-7a	/	[18]
	↑	Oncogene	Proliferation	FEN1		[66]
Lung cancer	/	/	Growth, invasion, cell cycle	miR-485-5p	/	[95]
HNSCC	↑	Oncogene	Scavenging and degradation, synthesis and metabolism, cell growth, death and motility	/	/	[67]
PDAC	↑	Oncogene	Aerobic glycolysis, proliferation	/	GLUT1	[68]
	↑	Oncogene	Cell growth	miR-141	PI3K/Akt	[20]
	↑	Oncogene	Proliferation, stemness-like properties		LncRNA DANCR	[70]
GBC	↑	Oncogene	Tumor growth	/	/	[71]
ERMS	/	/	Survival and growth	HMGA2	NRAS	[85]

↑: indicate up-regulated

*AML* acute myelocytic leukemia, *RAF1* rubisco assembly factor 1, *SOX2* SRY (sex determining region Y)-box 2, *GLUT1* glucose transporter 1

### IGF2BP2 in different cancers

#### IGF2BP2 in breast cancer

**Fig. 3:**
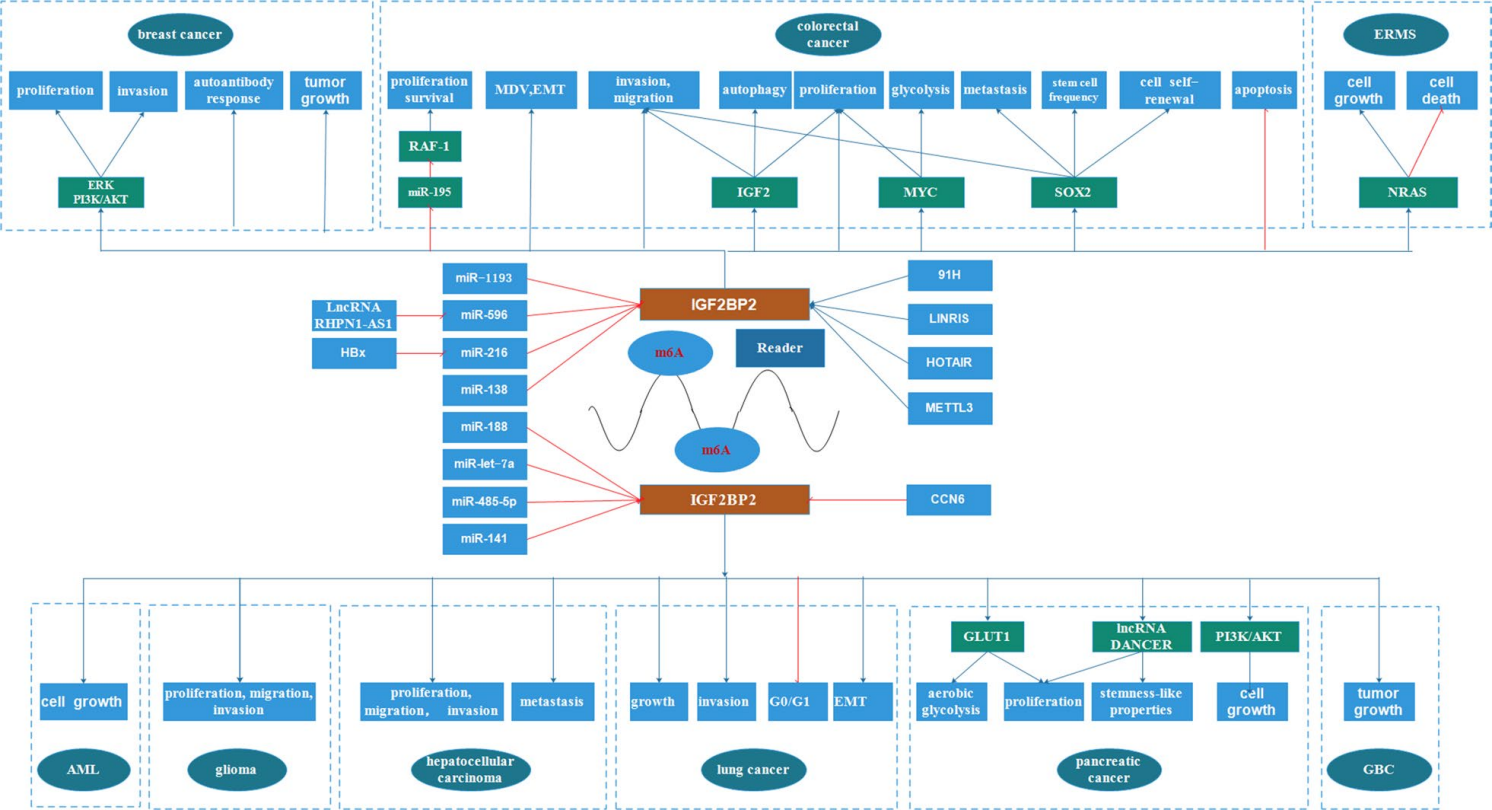
The role of IGF2BP2 in regulating multiple human cancer parameters. IGF2BP2 regulates proliferation, apoptosis, cell cycle, migration, invasion, cell viability, MDV, EMT, glycolysis, autophagy, self-renewal, tumor growth etc., of multiple cancer types such AML, breast cancer, colorectal cancer, ERMS, glioma, hepatocellular carcinoma, lung cancer, pancreatic cancer and GBC. IGF2BP2 performs its modulatory functions by interacting with miRNAs, lncRNAs, mRNAs and other m6A-related genes

IGF2BP2 rs4402960 increases the risk of developing breast cancer in female Chinese Hans [72]. Compared to luminal or apocrine subtypes, IGF2BP2 is over-expressed in basal-like breast cancer tissues [14]. Liu et al. [73] further reported that IGF2BP2 is over-expressed in breast cancer tissues, where it up-regulates auto-immune response. Consequently, over-expression of IGF2BP2 is not only a potential biomarker for developing breast cancer but also a novel diagnostic factor for the same disease. Meanwhile, Ccn6, secreted by normal breast epithelium, can suppress the expression of IGF2BP2 protein in cancerous breast tissues, thus modulating the growth of the tumor. Ccn6/Wisp3 knockdown up-regulated the expression of IGF2BP2 in mice, who developed mammary carcinomas characterized by spindle and squamous differentiation, validated hallmarks of metaplastic breast carcinomas [74] (Fig. 3).

#### IGF2BP2 in pancreatic cancer

IGF2BP2 is also over-expressed in PDAC [68, 69]. Meanwhile, Glucose transporter 1 (GLUT1) is an integral membrane protein consisting of 12 transmembrane helices and an intracellular domain which promotes aerobic glycolysis and proliferation of cancer cells [75–77]. IGF2BP2 also promotes aerobic glycolysis and proliferation of PDAC cells by directly binding to and stabilizing GLUT1 mRNA [68]. Further correlation analyses have revealed that over-expression of IGF2BP2 inhibits the expression of apoptosis (B-cell lymphoma-extra large) and ubiquitination (E3 ubiquitin ligase Smurf1 and F-Box protein 45) associated genes. Expression of IGF2BP2 also promotes tumor progression by inducing epithelial-mesenchymal transition (EMT).

#### IGF2BP2 in esophageal cancer

Multivariate logistic analyses have demonstrated that IGF2BP2 rs1470579 and rs4402960 polymorphisms increase the risk of developing esophageal squamous-cell carcinoma [78]. In contrast, a separate research showed that IGF2BP2 rs1470579 phenotype decreases the risk of developing esophagogastric junction adenocarcinoma in Eastern Chinese Han population [79]. Elsewhere, IGF2BP2 polymorphism has been found to increase the risk of developing human esophageal adenocarcinoma and Barret’s esophageal tissue. It also promotes growth, proliferation, metabolism and inflammation of cancer tissues [16].

#### IGF2BP2 in other cancers

Stratified analyses and haplotype analysis revealed that in Eastern Chinese Han population, IGF2BP2 rs1470579 and rs4402960 polymorphism decreased the risk of developing NSCLC among females < 60 years and non-alcohol drinker [19]. However, IGF2BP2 rs4402960 and rs6769511 phenotypes strongly predict (positive) response of metastatic gastric cancer patients to chemotherapy [80].

Over-expression of IGF2BP2 in AML patients negatively correlates with expression of CCAAT/enhancer binding protein α, a positive prognostic factor. Conversely, over-expression of IGF2BP2 positively correlates with the expression of poor prognostic factors including mutated FMS-like tyrosine kinase 3 and isocitrate dehydrogenase 1 [65]. Intriguingly, AML cells continue to grow in IGF2BP2 knockdown subjects. Expression of IGF2BP2 has also been shown to be up-regulated in colorectal cancer (CRC) tissues, promoting proliferation and survival of the cancer cells [81]. Analysis of The Cancer Genome Atlas (TCGA) data combined with immunohistochemical (IHC) tests [67] revealed that IGF2BP2 is over-expressed in HNSCC tissues, promoting scavenging and degradation, synthesis and metabolism and growth of tumor cells. In addition, over-expression of IGF2BP2 is a risk factor for poor prognosis of HNSCC patients. Besides, IGF2BP2 is frequently up-regulated in GBC, and has been shown to promote growth of xenograft tumors in mice. Moreover, over-expression of IGF2BP2 promotes the production of reactive oxygen species and expression of small GTPase Ras-related C3 botulinum toxin substrate 1 in GBC [71]. In addition, high mobility group AT-hook 2 (HMGA2), a DNA-binding protein, is often reactivated in various cancers. Expression of HMGA2 enhances metastasis and is associated with poor prognosis of cancers [82–84]. Interestingly, HMGA2 regulates IGF2BP2, a noble rhabdomyosarcoma (ERMS) protein key in survival and growth of cells. IGF2BP2 binds NRAS mRNA, regulating the expression of NRAS protein [85]. RPSAP52, an antisense transcribed pseudogene of HMGA2, promotes proliferation of sarcoma and self-renewal pathways by cross-linking with IGF2BP2 [86]. Except for HMGA2, IGF2BP2 markedly promotes functions of IGF and proliferation of cancer cells by binding and stabilizing HMGA1 [8].

### Mechanism underlying IGF2BP2 regulation of cancers

Mechanistically, IGF2BP2 modulates proliferation, migration, invasion, metastasis and apoptosis of cancer cells by regulation transcription of miRNAs, lncRNAs and other m6A-related genes [7, 9, 15].

#### IGF2BP2 with miRNAs in cancers

miRNAs are a group of endogenous, highly conserved, noncoding RNAs (18–25 nts in length) that regulate gene expression both transcriptionally and post-transcriptionally [87–90]. Accumulating evidence has demonstrated remarkable relationship between the expression patterns of miRNAs and IGF2BP2 and development as well as progression of tumors. For instance, miR-1193 is often down-regulated in breast cancer tissues and culture cell lines. However, over-expression of miR-1193 inhibits proliferation and invasion of breast cancer cells by binding the 3′UTR region of IGF2BP2 mRNA, activating ERK and PI3K/Akt signaling pathways [7]. The expression of IGF2BP2 is also up-regulated in CRC tissues, where it promotes proliferation and survival of the cancer cells by post-transcriptionally inhibiting miR-195-mediated degradation of rubisco assembly factor 1 [81]. Additionally, miR-188 is down-regulated in glioma cells and tissues, its over-expression inhibits proliferation, migration and invasion of glioma cells and tissues by directly targeting IGF2BP2 [91]. Specifically, miR-138 represses expression of IGF2BP2 by targeting its 3ʹ-UTR. This intern inhibits EMT and suppresses proliferation and invasion of low-grade glioma cells [17]. miR-216b also suppresses proliferation, migration and invasion of HCC by down-regulating the expression of IGF2BP2, found to be most often over-expressed in HCC tissues [92]. On the other hand, flap endonuclease-1 (FEN1), a multifunctional structure-specific nuclease critical in maintaining normal cell growth, is up-regulated in HCC [93, 94]. Pu et al. [66] reported that over-expression of IGF2BP2 promotes proliferation of HCC both in vitro and in vivo. Mechanistically, IGF2BP2 directly binds the m6A site on FEN1 mRNA, stabilizing the mRNA. Hepatitis B virus suppresses p53-mediated activation of miR-216b and promotes the expression of IGF2BP2. Furthermore, the expression of miR-let-7a which positively correlates with hypermethylation of MIRLRT7A3, modulates the expression of IGF2BP2 [18]. In lung cancer, the over-expressed miR-485-5p inhibits growth and invasion of cancer cells, arrests the G0/G1 cycle and disrupts the TGF-β-induced EMT by directly targeting IGF2BP2 [95]. Finally, the upregulated expression of IGF2BP2, a target for miR-141, promotes proliferation of PDAC via the PI3K/Akt signaling pathway [20].

#### IGF2BP2 and lncRNAs in cancers

LncRNAs, previously thought to cause transcriptional noise, are a class of non-protein-coding RNAs longer than 200nt that regulate several physiological and pathological processes [96, 97]. Increasing evidence shows that IGF2BP2, in conjunction with multiple lncRNAs, regulate multiple biological functions. For instance, lncRNA HOX transcript antisense RNA (HOTAIR) regulates the expression of target genes by directly interaction with histone modification complexes [98, 99]. IGF2BP2-mediated over-expresssion of HOTAIR promotes proliferation, migration, invasion, microvessel density value (MDV) and EMT, but represses apoptosis of colon cancer cells [9]. Moreover, lncRNA LINRIS inhibits K139 mediated ubiquitination of IGF2BP2, preventing the degradation of IGF2BP2 via autophagy-lysosome pathway [100]. Consequently, LINRIS knockdown weakens downstream effects of IGF2BP2, particularly MYC-mediated glycolysis in CRC cells and proliferation of cancer cells. On the other hand, lncRNA 91H, a long non-coding antisense transcript located at H19/IGF2 locus, participates in tumor development [101, 102]. lncRNA 91H silencing modulates proliferation, migration, invasion, autophagy and expression of mammalian target of rapamycin (mTOR) in CRC cancer cells by suppressing IGF2 expression, which up-regulates the expression of IGF2BP2 [103]. lncRNA RHPN1-AS1 promotes proliferation and metastasis but inhibits apoptosis of HCC cells [104]. lncRNA 91H performs its modulatory function via miR-596, which binds to IGF2BP2.

Intriguingly, research shows that lncRNAs and IGF2BP2 can regulate each other. For instance, IGF2BP2 promotes proliferation and stemness-like properties of pancreatic cancer cells by binding and stabilizing m6A modified DANCR RNA [70].

#### IGF2BP2 with other m6A-related genes in cancers

Further molecular insights implicate m6A alterations in the pathogenesis and development of cancers via regulating the expression of multiple tumor-associated genes [105, 106]. Besides, different m6A-ralated genes cross-link with each other to modulate the development of multiple cancers [107, 108]. METTL3 predominantly catalyses m6A methyltransferase system and regulates numerous processes in multiple human cancers [109]. METTL3 promotes self-renewal of CRC cell, proliferation and migration of stem cells in vitro as well as tumorigenesis and metastasis of advances CRC in vivo, mainly by targeting sex determining region Y (SRY)-box 2 (SOX2) [15]. However, METTL3 functions are IGF2BP2-dependent, which recognizes the coding sequence regions of methylated SOX2 transcripts, and prevents degradation of SOX2 mRNA.

## Discussion

This review describes the role and specific expression patterns of IGF2BP2 in human metabolic diseases and cancers. Even though IGF2BP2 SNPs are widely associated with the risk of developing diabetes, the relationship between expression patters of the resultant proteins and human metabolic diseases and cancers vary among ethnic populations. For instance, even though Grarup et al. [21] found no association between IGF2BP2 gene variants and T2D in Danish population, Groenewoud et al. [22] reported that expression of IGF2BP2 polymorphisms decreased glucose-stimulated insulin secretion in the first phase of diabetes development in Dutch and Germany’s population. More intriguingly, the association between IGF2BP2 SNPs and metabolic diseases varies even within the same ethnic population. For example, Zhang et al. [30] found that IGF2BP2 rs4402960 is associated with T2D in patients from Anhui, a province in China. However, the relationship between IGF2BP2 rs4402960 expression and T2D was insignificant in participants from Shanghai, China [50]. Hence, the associations between IGF2BP2 variations and T2D should be interpreted with caution. However, IGF2BP2 may induce human metabolic diseases via posttranscriptional regulation of various genes associated with specific cell types and pathways. For example, hepatocyte-specific IGF2BP2 knockdown inhibits oxidation of fatty acid, leading to accumulation of triglyceride in mice liver [13]. On the other hand, over-expression of IGF2BP2 increases the risk of developing numerous cancers. However, certain IGF2BP2 gene variants decrease the risk of NSCLC among females of Chinese Han population [19]. This suggests that the role of IGF2BP2 in cancers development varies among tumors and ethnic groups.

Ning Dai [4] had reviewed and summarized the expression of IGF2BP2 impairs insulin secretion. In addition, IGF2BP2 regulates multiple biological processes post-transcriptionally. Additionally, IGF2BP2 regulates multiple physiological processes including embryonic development, neuronal differentiation and metabolism, insulin resistance in diabetics and carcinogenesis [5]. Ning Dai focused on the associations and mechanism underlying expression of IGF2BP2 SNPs and the development of metabolic diseases including T2D, nonalcoholic steatohepatitis, obesity and fatty liver disease. We also summarized current works on the association among IGF2BP2, miRNAs, lncRNAs, mRNAs and other m6A-related genes and development as well as regulation of cancers. Findings of this research may uncover new frontier for the exploration of development and treatment of tumors.

Recent researches have focused on the role of metabolic pathways in various cancer parameters. Specifically, cancer cells exhibit metabolic reprogramming such as dysregulated glucose uptake, excessive lipid synthesis and glutaminolysis [110]. These transformations are essential parameters in the maintenance and development of malignant phenotypes in harsh microenvironments [111–115]. The role of IGF2BP2 in glucose tolerance, insulin sensitivity, fatty acid oxidation and the development of metabolic diseases had been reviewed recently [13, 21–23, 28, 53]. The metabolic role of IGF2BP2 in cancers is scarce. However, available evidence demonstrates that IGF2BP2 targets GLUT1, promoting aerobic glycolysis and proliferation of PDAC cells [68, 75–77]. IGF2BP2 also promotes metabolism of esophageal cancer and HNSCC [16, 67].

High-throughput sequencing technology recently revealed that m6A modification, circRNAs, miRNAs and lncRNAs are emerging important regulators of several biological process [116]. Overall, we speculate the complex relationship among IGF2BP2, circRNAs, miRNAs and lncRNAs participates in the development of both metabolic diseases and cancers. Nonetheless, further researches are needed to unlock the precise interrelationships among pathways regulated by the above molecules.

## Conclusion

The interrelationship among IGF2BP2, miRNAs, lncRNAs and their target genes with regard to cancers and metabolic diseases are reported but inconclusive. Nevertheless, current available evidence suggests the critical role of IGF2BP2 SNPs in the development of the two diseases.

## Data Availability

Not applicable.
